# Disseminated miliary lupus on the face: successful treatment with oral dapsone and topical metronidazole

**DOI:** 10.1016/j.abd.2025.501275

**Published:** 2026-01-19

**Authors:** Tania Rita Moreno de Oliveira Fernandes, Itamar Santos, Luciana Lócio Rosado, Pâmela Layra Laguna Dias, Luana da Silva Souza

**Affiliations:** a Department of Medical-Surgical Pathology of the Integumentary System, Universidade Federal do Vale do São Francisco, Petrolina, PE, Brazil; b Private Practice, Petrolina, PE, Brazil; c Graduation, Universidade Federal do Vale do São Francisco, Petrolina, PE, Brazil

Dear Editor,

Lupus miliaris disseminatus faciei (LMDF) is a rare granulomatous dermatosis of which the etiology is not yet fully elucidated, characterized by the appearance of erythematous papules on the central region of the face, although cases of extrafacial involvement have been reported.^1^ Its pathogenesis remains uncertain, and its relationship with chronic inflammatory processes and hypersensitivity reactions is being discussed.^2^, ^3^

LMDF predominantly affects young adults, with a slight predominance in males, and has a self-limiting course, and may leave residual atrophic scars. Histopathologically, it shows epithelioid cell granulomas with central necrosis and surrounding lymphocytic infiltrate, including multinucleated giant cells. However, the histological pattern may vary depending on the stage of the disease.^4^ Due to clinical and histological similarities, LMDF can be confused with other facial granulomatous dermatoses, such as granulomatous rosacea and sarcoidosis. The presence of caseous necrosis in granulomas is a distinctive element that aids in diagnostic differentiation.^5^, ^6^

The objective is to present a clinical case of LMDF in a male patient, highlighting its clinical manifestations, complementary examinations, and response to treatment, emphasizing the main diagnostic and therapeutic challenges of the condition.

A 49-year-old man came to the office presenting with multiple micropapules on the periocular, nasolabial, glabella, and malar regions, symmetrically distributed, sometimes with pustules (Figure 1, Figure 2), without diffuse redness, desquamation, or associated telangiectasias. In addition, recent endoscopic examinations showed moderate antral enanthematous gastritis, erosive esophagitis, intramucosal lymphoid follicles in the sigmoid colon and rectum, as well as active chronic ileitis - unrelated to the dermatological disease, according to the literature. The condition began approximately six months before, and there was a prior diagnosis of discoid lupus, according to the anatomopathological report.

**Figure 1:**
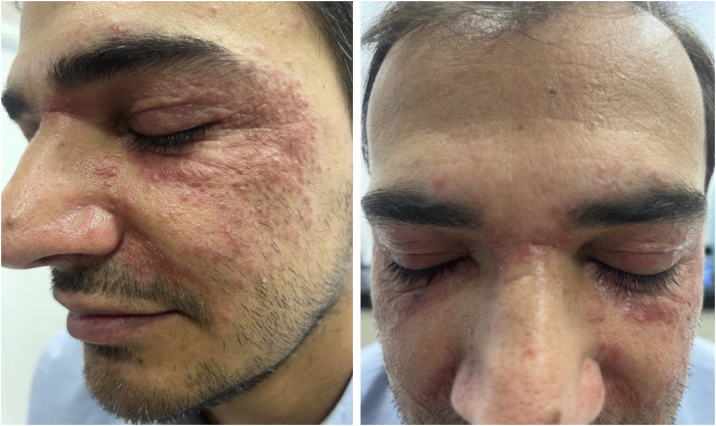
Multiple small erythematous papules, mostly grouped in plaques, on an erythematous base, on the periocular, nasolabial, and malar regions, symmetrically distributed. Photographs obtained with a smartphone.

**Figure 2:**
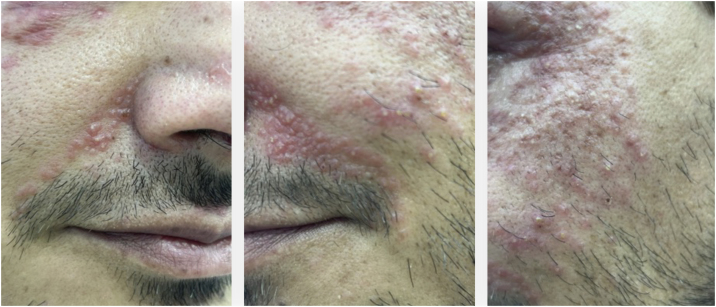
Close-up view of the skin lesions, showing hundreds of papules clustered on an erythematous background and some pustules. Photographs taken with a smartphone.

Initial treatment included hydroxychloroquine on alternate days, topical pimecrolimus, moisturizers, and sunscreen. After six months, even with the addition of oral corticosteroids and topical tacrolimus, there was no significant improvement. A new biopsy was performed, with suspicion of periorificial dermatitis or LMDF. The anatomopathological examination revealed epithelioid cell granulomas, some with central caseous necrosis and lymphohistiocytic infiltrate with giant cells (Fig. 3). Tests for fungi and AFB (PAS and Fite-Faraco staining) were negative, confirming the hypothesis of LMDF.

**Figure 3:**
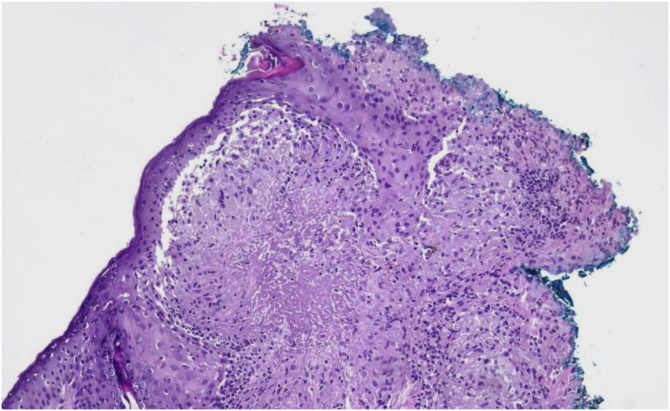
Skin sections show epidermis with hypergranulosis. The superficial dermis shows granulomas of epithelioid cells, some with central areas of necrosis in a caseous pattern and surrounding lymphohistiocytic infiltrate with multinucleated giant cells. (Hematoxylin & eosin, ×40).

Treatment was initiated with minocycline 100 mg/day and prednisone 20 mg/day, with gradual reduction of corticosteroid therapy over 15 days, in addition to topical metronidazole. After 30 days without a satisfactory response, it was decided, in agreement with the gastroenterologist (consulted at the patient's request), to introduce dapsone 100 mg/day orally, while maintaining topical metronidazole. By the twentieth day, a significant response was observed, with near resolution after four months (Fig. 4).

**Figure 4:**
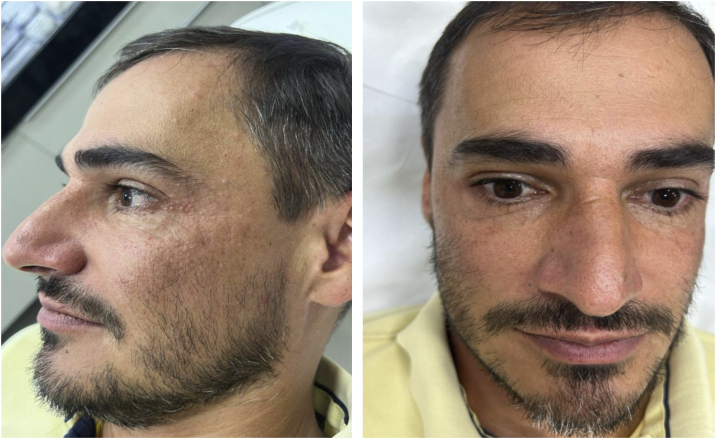
Patient after 4 months of treatment.

Dapsone is a dihydropteroate synthetase inhibitor drug with anti-inflammatory action. It has been used since the 1940s in the treatment of leprosy and various dermatoses, including granuloma annulare and dermatitis herpetiformis. Its potential adverse effects include methemoglobinemia, agranulocytosis, hepatitis, peripheral neuropathy, and hemolytic anemia, none of which were observed in the patient.^7^

Several systemic treatments are described as effective in LMDF: isotretinoin, dapsone, corticosteroids, clofazimine, metronidazole, topical tacrolimus, diode laser (1450 nm), and non-ablative fractional laser (1565 nm). Early use of low doses of corticosteroids can prevent scarring.^8^ The present case had results similar to those described by Patel et al.,^6^ in a 51-year-old woman, and Zawar et al., in a 23-year-old man treated with dapsone 100 mg/day for four weeks.^5^

In view of the above, it is concluded that the accurate diagnosis and adjusted therapeutic approach were fundamental for the management of LMDF. Considering the rarity of the condition and the slow response to conventional treatments, the combination of oral dapsone (100 mg/day) with topical metronidazole demonstrated efficacy and safety, highlighting the importance of a personalized strategy and continuous follow-up to optimize clinical results and minimize aesthetic and functional impacts.

## ORCID ID

Itamar Santos: 0000-0001-5288-4846

Luciana Lócio Rosado: 0009-0005-5862-3045

Pâmela Layra Laguna Dias: 0009-0009-0923-283X

Luana da Silva Souza: 0009-0009-6152-9550

Tania Rita Moreno de Oliveira Fernandes: 0000-0002-7061-2825

## Research data availability

Not applicable.

## Financial support

None declared.

## Authors' contributions

Tânia Rita Moreno de Oliveira Fernandes: Design and planning of the study; analysis and interpretation of data; intellectual participation in the propaedeutic and/or therapeutic conduct of the studied cases; critical review of the literature; approval of the final version of the manuscript.

Luciana Lócio Rosado: Design and planning of the study; collection, analysis and interpretation of data; intellectual participation in the propaedeutic and/or therapeutic conduct of the studied cases; critical review of the literature; approval of the final version of the manuscript; article reviewer.

Itamar Santos: Design and planning of the study; analysis and interpretation of data; intellectual participation in the propaedeutic and/or therapeutic conduct of the studied cases; critical review of the literature; approval of the final version of the manuscript.

Pâmela Layra Laguna Dias: Design and planning of the study; analysis and interpretation of data; intellectual participation in the propaedeutic and/or therapeutic conduct of studied cases; critical review of the literature; approval of the final version of the manuscript.

Luana da Silva Souza: Design and planning of the study; analysis and interpretation of data; intellectual participation in the propaedeutic and/or therapeutic conduct of the studied cases; critical review of the literature; approval of the final version of the manuscript.

## Conflicts of interest

None declared.
